# Contests versus Norms: Implications of Contest-Based and Norm-Based Intervention Techniques

**DOI:** 10.3389/fpsyg.2017.02046

**Published:** 2017-11-23

**Authors:** Magnus Bergquist, Andreas Nilsson, André Hansla

**Affiliations:** Department of Psychology, University of Gothenburg, Gothenburg, Sweden

**Keywords:** goals, contest, norm, intervention, pro-environmental behaviors

## Abstract

Interventions using either contests or norms can promote environmental behavioral change. Yet research on the implications of contest-based and norm-based interventions is lacking. Based on Goal-framing theory, we suggest that a contest-based intervention frames a gain goal promoting intensive but instrumental behavioral engagement. In contrast, the norm-based intervention was expected to frame a normative goal activating normative obligations for targeted and non-targeted behavior and motivation to engage in pro-environmental behaviors in the future. In two studies participants (*n* = 347) were randomly assigned to either a contest- or a norm-based intervention technique. Participants in the contest showed more intensive engagement in both studies. Participants in the norm-based intervention tended to report higher intentions for future energy conservation (Study 1) and higher personal norms for non-targeted pro-environmental behaviors (Study 2). These findings suggest that contest-based intervention technique frames a gain goal, while norm-based intervention frames a normative goal.

## Introduction

Goals are important in guiding peoples’ behavior and choices. To have a goal coupled with a certain behavior creates meaning and motivation. Still, our goals are not always articulated, deliberate, or even conscious. Rather, situational cues are often important to activate a certain goal (Bargh et al., 2001; Lindenberg and Steg, 2007). In this way, cues in our environment may also lead to behavioral change. When it comes to pro-environmental behaviors, change can be motivated by different goals. For example, hedonic, financial *or* pro-environmental goals may motivate energy saving (Schultz et al., 2015; Steinhorst et al., 2015), carpooling (Evans et al., 2013), or choosing eco-labeled food (Wier and Calverly, 2002). While at first glance a particular pro-environmental behavior may seem to be executed in the same way regardless of the underlying goal, depending on the goal, different behavioral as well as psychological consequences would be implied (Lindenberg and Steg, 2007; Bolderdijk et al., 2012; Steg et al., 2014). Similarly, interventions may be designed to target different motivational bases for encouraging pro-environmental behavior (Steg and Vlek, 2009; Abrahamse and Steg, 2013; Schultz, 2014). The present studies examine behavioral and psychological implications of two intervention techniques: the contest-based intervention and the norm-based intervention.

We define the contest-based intervention as a situation where individuals’ goal achievements are negatively correlated (Stanne et al., 1999), that is, individuals inhibit each other’s goal achievements in competitive situations (Deutsch, 1949). In a contest-based intervention, behavioral engagements are motivated by the incentive to win and individuals use other people as referents to be outperformed. In contrast, the norm-based intervention seeks to make social norms salient by showing other people’s behaviors and (dis)approvals (e.g., Schultz et al., 2007). In a norm-based intervention, behavioral change is motivated by the goal to adjust ones behavior to others, that is, to act “as others” or to do what one “ought to” (Deutsch and Gerard, 1955; Cialdini et al., 1990) As such, normative influence has metaphorically been described as a navigation tool in decision making, guiding people toward acting socially appropriately (Morris et al., 2015).

### Goal-Framing Theory

When people engage in controlled actions cognitive resources will be scarce. Therefore, beliefs and motivational processes will be structured in either the foreground or background of attention (Bargh, 1994; Lindenberg, 2000). Goal framing theory (GFT; Lindenberg, 2000, 2001; Lindenberg and Steg, 2007) predicts that a foreground (i.e., focal) goal will mobilize corresponding beliefs and motivations (Lindenberg, 2001; Steg et al., 2016). For example, when making a consumer decision in the local grocery store, a goal to feel good, to save money, or to act appropriately may direct our attention to the product’s taste, price, or ethical dimensions, respectively. A focal goal will thus influence accessibility of knowledge, information detection, perceived action alternatives, and affect how people act in a specific situation (Steg et al., 2014).

Goal framing theory identifies three overarching goals: (1) the hedonic goal activating pleasure seeking, (2) the gain goal activating the goal to protect or improve one’s resources, and (3) the normative goal sensitizing people to what one ought to do (Lindenberg, 2001). Drawing on GFT, we propose that a contest-based intervention frames a gain goal, motivating pro-environmental behaviors by improving one’s own profit. In contrast, a norm-based intervention will frame the normative goal, motivating pro-environmental actions by activating peoples’ goal to do “what one ought to.”

### Goal-Framing in Interventions

We propose that contest-based and norm-based interventions can be thought of as two forms of situational goal-framing processes. A contest-based intervention would frame a gain goal, leading people to construe the targeted pro-environmental behavior primarily in terms of own gain. In contrast, a norm-based intervention would frame a normative goal, leading people to construe the targeted pro-environmental behavior primarily in terms of “appropriateness” and “oughtness” (Lindenberg and Steg, 2007, 2013; Keizer et al., 2008, 2013; Steg et al., 2016). Although GFT does not distinguish between social and personal norms of pro-environmental behavior, here we argue that norm-based interventions typically utilizing social norms (e.g., descriptive and/or injunctive social norms, described in more detail in the next subsection) have implications also for personal norms. As such, a preexisting personal norm might be activated by communicating social norms assuming the norms overlap (see e.g., Verplanken and Holland, 2002). Alternatively, if an individual lacks such a personal norm, a social norm intervention might still foster a personal norm, tentatively via self-perception and internalization processes. In other words might a norm-based intervention in that case initiate pro-environmental behavior via external norm pressure that, once performed, initiates the development of an internal sense of oughtness (see e.g., Venhoeven et al., 2016). Based on GFT, we suggest that the norm-based intervention is a form of situational normative goal-frame activating personal norms and promoting motivation for long-termed pro-environmental behavior.

In contrast, a contest-based intervention will not activate such feelings of oughtness of pro-environmental behavior or motivation for long-termed effects. Hence, the mere performance of and engagement in a gain-seeking pro-environmental behavior would not feed back into and reinforce such feelings of oughtness (although it would reinforce gain seeking). In line with these expectations, past research has shown that priming people with money decreases pro-social behaviors (Vohs et al., 2006, see Vohs, 2015, for a review), possibly because money-priming induces a gain goal which pushes away normative obligations. As a consequence of framing a gain goal, motivating people to maximize own gains, we expect people to show intensive engagement in a contest-based intervention. However, the gain goal provides an unstable basis for stimulating pro-environmental engagement, as people will engage in the targeted behavior only as long as engagement is associated with improving one’s own gains (Steg et al., 2014; Alberts et al., 2016). Therefore, the contest-based intervention is not expected to promote motivation to conduct the targeted pro-environmental behavior in the future nor to promote motivation to conduct non-targeted pro-environmental behaviors.

#### Contests

##### Behavioral consequences of contests

Although practitioners have often used contests in pro-environmental intervention campaigns, to our knowledge, few empirical studies have evaluated contest-based interventions, and no past study has tested the psychological effect of contest-based interventions (e.g., Dwyer et al., 1993; Abrahamse et al., 2005; Fischer, 2008; Osbaldiston and Schott, 2012; Abrahamse and Steg, 2013; Schultz, 2014). Past research has found that contest-based interventions can promote energy-saving both in laboratory and in field experiments (Reeves et al., 2015). For instance, McClelland and Cook (1980) found that individuals engaging in a contest targeting energy conservation decreased their energy consumption by 9.8% during the first week and by 3.9% in the final sixth week during the intervention. A recent study comparing a contest-based intervention with a norm-based intervention showed that both interventions promoted energy-saving, but no long-term effect was found in the contest (Alberts et al., 2016).

##### Psychological consequences of contests

Contest-based interventions could be described as a specific form of economic incentive (Schultz, 2014), where monetary prizes are often used to motivate behavioral change (e.g., McClelland and Cook, 1980; Bornstein et al., 2002). Although economic incentives have been found to promote pro-environmental behaviors (Thøgersen, 2003; Bucciol et al., 2015; Maki et al., 2016), from a goal-framing perspective (Lindenberg, 2001; Lindenberg and Steg, 2007), economic incentives and contests are both intervention techniques that activate gain goals. The targeted behavior will therefore be framed so that people think and act in terms of improving or guarding own gains (see also Gneezy et al., 2011; Bolderdijk and Steg, 2015, for a discussion), which consequently weakens both normative obligations and long-term effects. In line with this reasoning, past research has found detrimental psychological effects of economic incentives, such as decreased internal motivation (Deci et al., 1999), crowded-out responsibility (Frey and Oberholzer-Gee, 1997; Gneezy and Rustichini, 2000), and reduced intent for pro-social behaviors (Festinger and Carlsmith, 1959; Mellström and Johannesson, 2008). In fact, mere exposure to money has been found to decrease altruism (Vohs et al., 2006; Gasiorowska et al., 2016; see Vohs, 2015). Finally, in line with our proposition, research has shown that contests per see decrease internal motivation (Reeve and Deci, 1996) and stimulate unethical behaviors (Kilduff et al., 2015) possibly because contests weaken feelings of obligation to conduct the targeted behavior.

#### Norms

##### Behavioral consequences of normative influence

The norm-based intervention have been used to promote pro-environmental behaviors such as recycling (Schultz, 1999), sustainable transportation (Kormos et al., 2014), decreased littering (Cialdini et al., 1990; De Kort et al., 2008), and to conserve both water (Schultz et al., 2016; Jaeger and Schultz, 2017) and energy in residential (Schultz et al., 2007, 2015; Nolan et al., 2008; Allcott, 2011) and public settings (Oceja and Berenguer, 2009; Bator et al., 2014; Dwyer et al., 2015; Bergquist and Nilsson, 2016). When setting up a norm-based intervention, studies have often included both injunctive social norms (information about whether other people approve or disapprove of the behavior) and descriptive social norms (information about what other people are doing) (Cialdini et al., 1990; Schultz et al., 2007; Smith et al., 2012; Hamann et al., 2015). Field-experiments have found that norm-based interventions cause both long-term effects (Hirayama, 2016; De Dominicis, 2017) and are more effective than monetary incentives (Schultz et al., 2015) and interventions appealing to concerns about the environment (Goldstein et al., 2008; Nolan et al., 2008). Drawing on GFT, we suggest that engaging in a norm-based intervention frames a normative goal, making people think about the targeted behavior in terms of obligations (e.g., personal norms), and making people motivated to future engagement in the targeted pro-environmental behavior.

##### Psychological consequences of normative influence

Although normative influence has been applied to change a number of pro-environmental behaviors, as reviewed above; few studies within environmental psychology have examined how normative influence affects cognitive processing. GFT predicts that when the normative goal is activated, for example by observing others’ norm-consistent behaviors, cognitive and motivational processes will be directed toward acting appropriately; strengthening moral obligations for pro-environmental behaviors (Lindenberg and Steg, 2007, 2013; Lindenberg, 2009). Such pro-environmental obligations are often measured by personal norm, which have shown to be predictive of various pro-environmental behaviors and related to individual’s value orientations (Nordlund and Garvill, 2002, 2003; Gärling et al., 2003; Steg et al., 2005; Thøgersen, 2006; Bamberg and Möser, 2007; van der Werff and Steg, 2015). Hence, normative goal-framing is expected to activate personal norms, defined as an internally sensed obligation to perform some specific or general pro-environmental behavior. In addition, given the long-termed effect of norm-based interventions (e.g., De Dominicis, 2017) and that framing environmental aspects of energy conservation positively affects non-targeted behaviors (e.g., Steinhorst and Matthies, 2016), the norm-based intervention is expected to promote motivation for long-termed engagement and activate personal norms for targeted as well as non-targeted behaviors.

### The Present Research

The aim of the present research was to compare behavioral and psychological effects of contest-based versus norm-based intervention techniques to promote pro-environmental actions. Study 1 tested if contest-based vs. norm-based intervention techniques differ in (1) level of engagement in the targeted behavior, (2) activation of personal norms and (3) intentions for future energy conservation. Study 2 complements Study 1 by controlling for level of engagement and the role of financial incentives. Furthermore, Study 2 targets virtual recycling behavior and also tests whether contest-based and norm-based intervention techniques influence personal norms for non-targeted pro-environmental behaviors.

## Study 1

The aim of Study 1 was to examine whether and how contest-based and norm-based intervention techniques promoting energy-saving behavior would influence personal energy-saving norms and behavioral intentions for future energy-saving. Given our methodological setup and because gain goals would promote intensive behavior, we first expected that participants in the contest-based intervention technique would more strongly engage in the targeted behavior (i.e., write more energy-saving tips) than participants in the norm-based intervention technique (H1). Moreover, as a consequence of the norm-based intervention technique framing a normative goal, we hypothesize that participants in the norm condition would express stronger personal energy saving norms (H2a) and stronger intentions for future energy conservation (H2b) than participants in the contest condition. Since the personal norms also reflect basic (stable) environmental values, it is reasonable to also assume personal norms to positively influence number of energy saving tips written (H3a) and intentions for future energy conservation (H3b) in both intervention techniques. In addition, since the norm-based intervention is predicted to activate normative considerations, we expect that the positive correlation between personal norms and number of energy saving tips written and intentions for future energy conservation could possibly depend on condition. Because environmental obligations should be framed by the normative goal, participants in the norm-based intervention is expected to more strongly act upon such obligations, therefore we hypothesize that participants in the norm-based intervention will show stronger correlations between personal norms and both number of energy saving tips written (H4a) and intentions for future energy conservation (H4b) than participants in the contest-based intervention.

### Method

#### Participants

One hundred and fifty individuals located in the United States participated in an experimental survey on *Amazons Mechanical Turk* (MTurk) during spring 2016 and were paid ¢50. MTurk is an online marketplace where employers are free to choose among various small tasks described by content and payment. In a between-subjects design, the software *Qualtrics* was used to randomly assign participants to a contest or a norm condition. All participants actively volunteered and signed up to conduct “*A survey in social psychology*” and were informed about the length, content and payment for conducting the task. Therefore respondents should be considered consciously aware of participation in general. All participants were given the opportunity to contact the first author via MTurk. All participants were informed that their participation would be treated anonymously and confidentially, used for research purposes only, and that they had the right to end their participation at any time.

#### Procedure and Design

All participants were provided with information on energy-saving comprising a text (461 words), three pictures, and one video. Participants were asked to spend 3 minutes writing as many energy-saving tips as possible based on the material, using 30 rows of empty text fields at the bottom of the page.

In the contest condition, participants were shown the text “*Let the competition begin!”* and informed that the person writing the most tips would be rewarded with ¢50 for each written tips (through the MTurks bonus system). The cumulative prize money, ranging from ¢50 to $15, were shown next to each row of empty text fields. In the norm condition, participants were exposed to an authentic injunctive energy-saving norm, demonstrated by a text and a graph depicting that approximately 90% of American MTurkers rated energy-saving as good^1^. The 30 rows were only labeled by the tip number ranging from “*tip number 1”* to “*tip number 30”* in the norm condition.

Finally, in both conditions, the seventh row provided participants with authentic information about other people’s behavior “*MTurkers write 7 tips on average.”* Thus, both conditions were provided with information showing how other people were expected to behave. We believed that this information would be interpreted as a descriptive norm for participants in the norm condition, while participants in the contest condition would interpret this information as a reference point that must be outmatched in order to win the prize.

#### Measures

After completing the task, participants answered four questions. Based on Thøgersen (2003), two items measured personal energy-saving norms: “*Do you feel obligated to save household energy as often as possible?”* rated from 1 (*No, no obligation*) to 9 (*Yes, very strong obligation*) and “*I think I ought to save household energy as often as possible”* rated from 1 (*Totally disagree*) to 9 (*Totally agree*) (*M* = 7.53, *SD* = 1.44). Two items measured behavioral intentions, based on items from past research (Smith et al., 2012) and Ajzen’s (2002) recommendations, stated as *“I intend to engage in energy conservation in the forthcoming month*,” and “*I will try to engage in energy conservation in the forthcoming month”* ranging from 1 (*very unlikely*) to 9 (*very likely*) (*M* = 7.71, *SD* = 1.49). Number of energy-saving tips written by each participant were counted and used as a measure of engagement in the targeted behavior (*M* = 10.57, *SD* = 6.09).

Because hedonism has been related to pro-environmental attitudes, choices, and self-reported behavior (Steg et al., 2012), a measure of hedonism was included in order to control for possible differences in hedonic goal framing (Lindenberg, 2000). Partially based on the hedonic values described by Schwartz (1992), all participants were presented with the statement *“To write energy-saving tips felt…,”* followed by five items semantically anchored on a 1*–*9 scale ranging through “*very unenjoyable – very enjoyable*; *very boring – very fun*; *very unpleasant – very pleasant*; *very hard – very easy*; and *very complicated – very uncomplicated”* (*M* = 6.51, *SD* = 1.62).

Four exclusion variables were included to remove former participants and participants who did not pay sufficient attention. All participants were asked whether they had conducted the present or a similar survey on energy-saving before and asked to report the average number of energy-saving tips written by other MTurkers and the number of minutes they were asked to spend on the task. Participants were also asked to report either the prize in the contest or the content of the injunctive norm depending on condition. Finally, participants filled in their gender, age, and were given the opportunity to leave a comment.

### Results

#### Exclusion and Reliability Analysis

The data contained four outliers, 20 incorrect answers to the three attention checks, three participants who did not write any energy-saving tips, and 21 individuals reporting former participation^2^. The final sample therefore consisted of 114 participants (54.5% males, *M*_age_ = 34.29, *SD* = 9.7). Cronbach’s alpha analyses showed acceptable values for items measuring personal norm (α = 0.82), behavioral intention (α = 0.94), and hedonism (α = 0.88).

#### Main Analysis

The conditions were first compared for ratings of hedonism to control for hedonic goal framing. Results showed a non-significant difference between the conditions (*p* = 0.26). As expected, participants in the contest condition (*M* = 13.79, *SD* = 7.27, *n* = 53) wrote more energy-saving tips than participants in the norm condition (*M* = 7.77, *SD* = 2.62, *n* = 61), *t*(63.66) = 5.7, *p* < 0.001, *d* = 1.13, 95% CI [0.74, 1.53], supporting H1. In testing H2a and H2b, number of energy saving tips and hedonism was used as covariates, and personal norms and intentions were compared between the conditions. Although the results were in the predicted direction, personal norms did not differ between conditions, *t*(112) = 1.39, *p* = 0.24, *d* = 0.23. In line with H2b, participants in the norm-based intervention showed a tendency for stronger intentions for future energy conservation [*t*(112) = 3.3, *p* = 0.07, *d* = 0.35]. In order to test H3 and H4, we conducted two hierarchical regression analyses, one for energy saving tips and the other for intention for future energy conservation, entering personal norm and intervention as the independent variables in the first step, and the interaction between the two in the second step. Results showed a main effect of personal norms, supporting H3a [β = 0.86, *SE_β_* = 0.34, *t*(110) = 2.53, *p* = 0.02]. However, the interaction was not significant [β = 0.32, *SE_β_* = 0.69, *t*(110) = 0.46, *p* = 0.65], rejecting H4a. In testing H3b and H4b, we ran the same moderation analysis, now entering intentions for future energy conservation as the dependent variable. A main effect of personal norms supported H3b [β = 0.91, *SE_β_* = 0.05, *t*(110) = 19.28, *p* < 0.001]. However, the interaction was not significant [β = 0.10, *SE_β_* = 0.10, *t*(110) = 0.11, *p* = 0.91], rejecting H4b.

### Discussion

First, Study 1 verified that the contest-based intervention technique promoted more intensive behavioral engagement (i.e., writing more energy saving tips) than a norm-based intervention technique. Although results were in the predicted direction, we found no overall effect of the intervention technique on personal norm. In line with H2b, a marginally significant effect showed higher intentions for future energy conservation for participants in the norm-based intervention technique. These results indicate that the contest-based intervention promoted intensive yet short-termed engagement, while the norm-based intervention promoted less intensive engagement but higher motivation for long-termed engagement. As hypothesized, the personal norm was overall positively correlated with writing energy saving tips and intention for future energy conservation. These positive relations were however not moderated by intervention technique, possibly suggesting that participants with strong norms did not lose motivation to engage intensively under contest condition, and that participants with weak personal norms in comparison still had a relatively weak motivation to engage intensively under a contest. The lack of support for H2a and H4 may be due to limitations in Study 1. First, the psychological effects of the contest-based and norm-based intervention technique were compared between groups that differed in level of engagement. Hence, number of energy saving tips written may have affected goal-framing. Therefore, Study 2 compared the contest-based and norm-based intervention techniques under equal levels of engagement. Second, economic incentives were confounded with the operationalization of the contest. That is, we do not know whether the behavioral and psychological effects of engaging in a contest-based intervention technique were due to the economic incentive or to participants’ engagement in a contest. Study 2 addressed this second shortcoming by explicitly using non-monetary incentives in the contest condition.

## Study 2

Because Study 2 was designed to compare conditions in which participants completed the task, our first purpose of Study 2 was to compare the conditions on speed and accuracy of task performance (Beersma et al., 2003). In Study 2, participants were told that the person completing the task fastest will win a prize. Thus, faster performance led to an increased chance of improving ones resources, in the contest condition only. Therefore, compared to the norm condition, we predict that participants in the contest condition will use less time to complete the recycling task (H5). Moreover, because increase working speed has been associated with making more errors (e.g., Beersma et al., 2003), it is predicted that participants in the contest condition will make more errors compared to participants in the norm condition (H6). Study 2 was also designed to compare personal norms between the two intervention techniques when participants engaged equally in the targeted pro-environmental behavior, now a fictive recycling task. Study 2 tested if the normative goal frame would activate personal norms for non-targeted behavior, as suggested by GFT (i.e., the cross norm effect showing that goal-framing spreads across behaviors; Keizer et al., 2008, 2013). There are both theoretical (Bolderdijk and Steg, 2015; Steg et al., 2016) and empirical (Steinhorst et al., 2015) reasons to expect that a normative goal frame could affect non-targeted behaviors. When assessing the impact of intervention techniques, effects on non-targeted behaviors are important because engaging in a first pro-environmental behavior could motivate people to also engage in other pro-environmental behaviors (e.g., Thøgersen and Noblet, 2012). On the other hand, when interventions target a specific pro-environmental behavior, people may stop acting pro-environmental in other domains (e.g., Chitnis et al., 2013; see Thøgersen and Crompton, 2009; Truelove et al., 2014; Nilsson et al., 2017 for review). Our hypotheses are that participants assigned to the norm-based intervention technique would express stronger personal norms for non-targeted behaviors, pro-environmental policy acceptance (H7, and energy-saving (H8) than participants in the contest condition.

### Method

#### Participants

Based on effect sizes found in our pre-studies, a power analysis using *G^∗^Power* (test family: *t*-test, difference between two independent means, two-tailed, effect size: *d* = 0.40, α = 0.05, β = 0.80), suggested a sample size of *n* = 200. Approximately, 30% of the participants were expected to be excluded due to failed attention checks and former participation. In a between-subjects design, 340 individuals were recruited to reach our necessary sample size. Participants, all located in the United States, conducted an experimental survey on MTurk during spring 2016, and were paid ¢30 for their participation. All participants actively volunteered and signed up to conduct “*A survey in social psychology*” and were informed about the length and content for conducting the task. Therefore respondents should be considered consciously aware of participation in general. All participants were given the opportunity to contact the first author via MTurk. All individuals were informed that their participation would be treated anonymously and confidentially, used for research purposes only, and that they had the right to end their participation at any time. Using Qualtrics, participants were randomly assigned to one of two experimental conditions.

#### Procedure and Design

Participants in the norm condition were exposed to an authentic injunctive recycling norm (similar to Study 1), a picture of people engaging in cooperation, and a text reading “*When we recycle together, we build a more sustainable society!”* In the contest condition, participants were informed that the recycling task was a contest, in which each item recycled was equal to one point, and that the individual who recycled all the items fastest would win a prize. Participants were also shown a picture of people engaging in a competition, and a text reading “*Get points fast and you can be the winner of a prize!”* To control for the potential influence of economic incentives, we did not specify the prize to participants (who were thus not aware of what it was). However, for practical reasons the prize was a monetary bonus provided via MTurk.

Participants were asked to drag and drop pictures of household waste to the correct recycling box. The task included 50 items (10 of glass, 14 of steel/aluminum, 13 of paper, and 13 of plastic) and four recycling boxes (*paper, glass, steel/aluminum*, and *plastic*). The items were labeled cumulatively, from “*1 point”* to *“50 points*” in the contest condition, and from “*Item 1”* to *“Item 50*” in the norm condition. After recycling all 50 items, participants in the contest condition were shown the text *“Good job! You have 50 points and can win the prize!”* In the norm condition, the final text read *“Good job! You have promoted sustainability for us all!”*

#### Measures

After completing the task participants were presented with measures of personal energy-saving norms (*M* = 7.42, *SD* = 1.50) and personal norms for accepting pro-environmental policies (*M* = 6.85, *SD* = 1.92), both including two items based on Thøgersen (2003; as used in Study 1). We expect that participants in the contest condition would use less time to complete the task than the norm condition, and consequently also make more errors (Beersma et al., 2003); therefore we also measured time and number of errors. To assess working speed, time spent on the page containing the recycling task was measured (*M*_sec_ = 109.21, *SD* = 28.32) and errors in the recycling task were measured by the number of items that participants dragged and dropped into an incorrect recycling box (*M* = 1.34, *SD* = 1.29). Study 2 also included a manipulation check asking participants “*How did the recycling task make you feel?”* rated on a scale from 1 (*Very cooperative*) to 9 (*Very competitive*). Finally, all participants were asked to fill in their gender, age, and were given the opportunity to leave a comment.

### Results

#### Exclusion and Reliability Analysis

Twenty-nine participants who did not complete the task were removed from the data: 13 outliers (two for errors, two for time, and nine for personal energy-saving norms), 25 participants answering incorrectly to at least one attention check (13 participants in the contest condition), and 69 individuals reporting former participation were also excluded^3^. The final sample consisted of 233 participants (56.2% males, *M*_age_ = 32.27, *SD* = 9.67, *n* = 107 in the contest condition). A Cronbach’s alpha reliability analysis showed acceptable values for personal norms for pro-environmental policy acceptance (α = 0.84) and energy-saving (α = 0.88).

#### Main Analysis

First, our manipulation check confirmed that conducting the recycling task in the contest condition made participants feel more competitive (*M* = 6.69, *SD* = 2.24, *n* = 107), while participants in the norm condition perceived the task as more cooperative (*M* = 4.03, *SD* = 2.45, *n* = 126), *t*(229.7) = 8.6, *p* = < 0.001, *d* = 1.13, 95% CI [0.85, 1.41]. As expected, participants in the contest condition completed the recycling task faster (*M*_sec_ = 104.50, *SD* = 27.28) than participants in the norm condition (*M*_sec_ = 113.25, *SD* = 28.68), *t*(230) = 2.4, *p* = 0.02, *d* = 0.31, 95% CI [0.05, 0.57], supporting H5. Also in line with H6, a marginally significant difference indicated that participants in the contest condition made more errors (*M* = 1.51, *SD* = 1.44) than participants in the norm condition (*M* = 1.19, *SD* = 1.13), *t*(199.91) = 1.87, *p* = 0.06, *d* = 0.25, 95% CI [-0.01, 0.51].

To test H7 and H8, two *t*-tests were conducted to compare the conditions on personal norms for pro-environmental policy acceptance and personal energy-saving norms. For pro-environmental policy acceptance, participants in the norm condition showed a tendency for stronger personal norms (*M* = 7.06, *SD* = 1.70) compared to participants in the contest condition (*M* = 6.60, *SD* = 2.14), *t*(201.02) = 1.85, *p* = 0.07, *d* = 0.24, 95% CI [-0.02, 0.50]. Likewise, participants in the norm condition showed a tendency for stronger personal energy-saving norms (*M* = 7.58, *SD* = 1.28) compared to those in the contest condition (*M* = 7.22, *SD* = 1.71), *t*(186.02) = 1.75, *p* = 0.08, *d* = 0.24, 95% CI [-0.02, 0.51]. Although H7 and H8 cannot be accepted at a conventional level of statistical significance, these results are in line with both hypotheses.

### Discussion

In Study 2 all our hypotheses derived from GFT followed the predicted pattern. When targeting recycling behaviors, participants in the contest-based intervention felt more competitive and worked faster, but also tended to make more errors than participants in the norm-based intervention. In the norm-based intervention, however, participants showed a tendency of expressing stronger personal norms for non-targeted pro-environmental behavior, suggesting that the norm-based intervention framed a normative goal. These findings corroborate those of Study 1, indicating that the contest-based intervention technique framed a gain goal, which directed participants’ cognitive processes toward winning; thus recycling was used as a means to improve participants’ own resources. In contrast, the norm-based intervention technique seemed to activate a normative goal, framing cognitive processes in terms of acting appropriately which subsequently promoted a tendency to make fewer errors and to feel more obliged to act pro-environmentally even outside the targeted behavior. It should however be noted that a control group would have been needed in order to infer the direction of these differences.

## General Discussion

Study 1 found that in the contest-based intervention technique, framing energy-saving as a means to make money, participants wrote more energy-saving tips than participants in the norm condition. Study 1 also indicated stronger intentions for future energy conservation in the norm-based intervention technique compared to the contest-based intervention technique, suggesting that the act of writing energy saving tips was more guided by instrumental motives for participants in the contest or/and more strongly framed as long-term for participants in the norm-based intervention. Study 2 further examined the consequences of contest-based versus norm-based intervention techniques, now targeting recycling. Results showed that the contest-based intervention technique prompted participants to work faster but also tended to make more errors. In line with GFT, these findings once again suggest that a contest-based intervention can promote high engagement in the targeted behavior. More interestingly, Study 2 showed that participants in the norm-based intervention technique tended to express stronger personal norms for non-targeted pro-environmental behavior. Although this is not a spillover effect, defined as the extent to which pro-environmental behavioral interventions promotes engagement in other non-targeted pro-environmental behaviors (e.g., Thøgersen and Crompton, 2009; Truelove et al., 2014; Nilsson et al., 2017), these findings complement previous research showing that personal norms may mediate or moderate the positive spillover effect (Thøgersen, 2004; Steinhorst et al., 2015). As such, the tendency to express stronger personal norms for non-targeted behavior in the norm-based intervention technique could be important both for understanding the psychological processes involved in spillover effects and also for practical considerations, suggesting that norm-based intervention techniques could serve as a basis for broader pro-environmental engagement. However, future research should further examine the tendency for norm-based intervention techniques to elicit positive spillover effects.

Taken together, the present research suggests that a contest-based intervention technique activates a gain goal, making people think and act upon the targeted behaviors in terms of how to guard or improve one’s own resources. In contrast, a norm-based intervention technique seems to frame a normative goal, leading people to think and act upon the targeted behavior in terms of “oughtness” and wishing to act appropriately. Our findings show that these two intervention techniques differ in both behavioral and psychological implications.

Adding to the integrated framework for encouraging pro-environmental behaviors (Steg et al., 2014), the present study corroborates goal-framing processes in contest-based and norm-based intervention techniques. This paper thus contributes to our applied understanding of goal-framing (Steg et al., 2016) and the scarce research on contest-based interventions (e.g., Abrahamse et al., 2005). In line with past research, our findings further highlights the limitations of using contests (Deci et al., 1981) and economic incentives in promoting pro-environmental behavior (Bolderdijk et al., 2012), but also showed that contests can promote intensive engagement in the targeted behavior. Moreover, adding to the research on normative influences (e.g., Schultz et al., 2007), the present research indicates that norm-based interventions may framing the normative goal, promoting motivation for long-term engagement and perceived obligations for non-targeted behaviors.

MTurk was used to recruit participants to the present studies. Recent research suggests that MTurk provides both good data quality, a better variety on demographic variables (Buhrmester et al., 2011; Casler et al., 2013), and more attentive participants than student pool participants (Hauser and Schwarz, 2015). Therefore, we argue that data in these studies had sufficient quality, and we attribute the data exclusions to our rigid attention checks, and because many individuals had participated in our pre-studies. Apart from the data quality as such, using MTurk samples does imply paying participants for taking our survey. Although the paid amounts are typically very small, in terms of goal-framing, this may have framed a gain goal for participants in both conditions (at least before they experienced the conditions). Given the effect on intention and non-targeted behaviors we may yet conclude that engaging in norm-based intervention techniques activate pro-environmental obligations even in contexts characterized by gain goals. It should also be noted that both studies followed ethical guidelines in Sweden for survey data and were thus conducted in line with the declaration of Helsinki.

Participants in the norm-based intervention did not report significantly higher personal norms than participants in the contest-based condition in Study 1. This null-finding could be due to an invalid measure of the normative goal. We used personal norms to measure normative goal-framing. However, since the personal norm is a relatively stable construct, this implies that it may only partially be affected by framing. Moreover, we expected goal-framing to result in change in reported *strength* of personal norms. This is only one possible implication of a normative goal-frame. Another possible implication is for example that a normative goal-frame will increase accessibility of normative considerations. As a second limitation, we did not distinguish between winning and losing the contest. Past research has found that winning a contest can increase intrinsic motivation (Reeve et al., 1985), which may promote future engagement in the targeted behavior. However, this possible increase in intrinsic motivation only applies to the winner and not for the majority of participants. It should also be noted that we examined ideal types of contests. As discussed in the GFT literature (Lindenberg and Steg, 2007; Steg et al., 2014), conflicts between the overarching goals may be reduced, as such contest-based interventions that does not push aside normative considerations may be designed. Future research should examine how this may be practically achieved. Furthermore, the normative feedback in Study 2 contained both social norms and sustainability related wordings. It cannot be ruled out that these wordings activated a distinct general value to act pro-environmentally. As such, the normative feedback may have been confounded by information that induced the normative goal per see. However, from a Goal-framing perspective, we argue that social norms and sustainable related wordings both frame the overarching normative goal. We encourage future research to examine how normative information should be used in framing the normative goal. Finally, although participants in the present study did not engage in actual energy-saving and recycling behaviors, resent research has demonstrated behavioral implications similar to our findings (Alberts et al., 2016).

Should an intervention targeting pro-environmental behaviors be contest-based or norm-based? Schultz (2014) argued that the efficiency of different intervention techniques depends on aspects of the targeted behavior. Furthermore, we suggest that different intervention techniques also lead to different psychological and behavioral implications. Therefore, a practical advice for decision-makers would be to match the psychological and behavioral implications of the intervention to the targeted behavior. Contest-based interventions may be used when aiming to promote intensive but short-term engagement while norm-based intervention techniques may be used when aiming to promote long-term effects and possibly as a basis for positive spillovers. In conclusion, it was found that a contest-based intervention technique stimulated strong and fast behavioral engagement, but it also seemed to discourage feelings of pro-environmental obligation. It was also found that a norm-based intervention technique tended to make participants more motivated to engage in future energy conservation and feeling more obliged to engage in non-targeted pro-environmental behaviors.

## Author Contributions

MB and AN developed the study concept and designed the Experiments. MB collected the data. MB and AH analyzed the data. All authors drafted the manuscript and approved the final version of the manuscript.

## Conflict of Interest Statement

The authors declare that the research was conducted in the absence of any commercial or financial relationships that could be construed as a potential conflict of interest.

## Footnotes

1 Based on a pre-study using a MTurk sample.

2 These exclusions do not represent unique participants but the number of data points for each control, therefore the number of exclusions is higher than the number of participants excluded.

3 These exclusions do not represent unique participants but the number of data points for each control, therefore the number of exclusions is more than the number of participants excluded.
